# Back to the basics: identifying positive youth development as the theoretical framework for a youth drug prevention program in rural Saskatchewan, Canada amidst a program evaluation

**DOI:** 10.1186/1747-597X-8-36

**Published:** 2013-10-22

**Authors:** Colleen Anne Dell, Charles Randy Duncan, Andrea DesRoches, Melissa Bendig, Megan Steeves, Holly Turner, Terra Quaife, Chuck McCann, Brett Enns

**Affiliations:** 1 Department of Sociology & School of Public Health, University of Saskatchewan, Saskatoon, Saskatchewan, Canada; 2 Department of Sociology, University of Saskatchewan, Saskatoon, Saskatchewan, Canada; 3 College of Medicine, University of Saskatchewan, Saskatoon, Saskatchewan, Canada; 4 School of Public Health, University of Saskatchewan, Saskatoon, Saskatchewan, Canada; 5 College of Education, University of Saskatchewan, Saskatoon, Saskatchewan, Canada; 6 Department of Psychology, University of Saskatchewan, Saskatoon, Saskatchewan, Canada; 7 Prince Albert Parkland Health Region, Prince Albert, Saskatchewan, Canada

**Keywords:** Youth drug prevention program, Outreach worker service, Rural school, Positive youth development, Program evaluation, 40 developmental assets

## Abstract

**Background:**

Despite endorsement by the Saskatchewan government to apply empirically-based approaches to youth drug prevention services in the province, programs are sometimes delivered prior to the establishment of evidence-informed goals and objectives. This paper shares the 'preptory’ outcomes of our team’s program evaluation of the Prince Albert Parkland Health Region Mental Health and Addiction Services’ Outreach Worker Service (OWS) in eight rural, community schools three years following its implementation. Before our independent evaluation team could assess whether expectations of the OWS were being met, we had to assist with establishing its overarching program goals and objectives and 'at-risk’ student population, alongside its alliance with an empirically-informed theoretical framework.

**Methods:**

A mixed-methods approach was applied, beginning with in-depth focus groups with the OWS staff to identify the program’s goals and objectives and targeted student population. These were supplemented with OWS and school administrator interviews and focus groups with school staff. Alignment with a theoretical focus was determined though a review of the OWS’s work to date and explored in focus groups between our evaluation team and the OWS staff and validated with the school staff and OWS and school administration.

**Results:**

With improved understanding of the OWS’s goals and objectives, our evaluation team and the OWS staff aligned the program with the Positive Youth Development theoretical evidence-base, emphasizing the program’s universality, systems focus, strength base, and promotion of assets. Together we also gained clarity about the OWS’s definition of and engagement with its 'at-risk’ student population.

**Conclusions:**

It is important to draw on expert knowledge to develop youth drug prevention programming, but attention must also be paid to aligning professional health care services with a theoretically informed evidence-base for evaluation purposes. If time does not permit for the establishment of evidence-informed goals and objectives at the start-up of a program, obtaining insight and expertise from program personnel and school staff and administrators can bring the program to a point where this can still be achieved and theoretical linkages made after a program has been implemented. This is a necessary foundation for measuring an intervention’s success.

## 

Here is an example of a 17 year old with no social supports, we are trying to set him up, he has no modeling at home for budgets, cleanliness, tidiness, meals, etc. He has no family life, and stays in the computer lab after school until it closes. He is waiting when I arrive in the morning. He only eats bagels as apparently this is the only food in his house. So he started in the breakfast program at the school. His caregiver, Grandma, usually spends her time in the local bar. He is not a user [of substances] yet but he needs coping skills. – Prince Albert Parkland Health Region School Principal, Interview #4, 2012.

## Background

It is well established that individuals who initiate the use of substances earlier in life have a greater likelihood that they will experience adverse consequences later in life, including addiction [1-3]. A combination of factors generally puts a youth, defined as 12–18 years of age, at-risk. Research has shown that the initiation of substance use is closely linked with social and environmental factors, whereas early initiation of use as well as abuse are highly linked to genetic and psychological determinants [2,4]. The determinants that place youth 'at-risk’ of problematic substance use are generally categorized [5] as individual (e.g., age, gender, neurophysiological vulnerability) [1,6-11], interpersonal (e.g., family, peers, school), [2,9,12-16] and social and cultural/community (e.g., media portrayals, social norms, street involvement) [5,17-21].

Exposure to risk factors alone does not determine youth involvement in drug use and abuse. Increasingly research is showing the benefits of protective factors; community assets and individual resiliency have been identified to mitigate the influence of risk factors [12,16,17,22,23]. According to the work of Saeywc (2007), “[c]onnectedness to school, positive relationships with caring adults within or outside of the family, and supportive peers seem to reduce the likelihood of the distress and difficulties in coping that lead to problem substance use” (18). A recent study by The McCreary Centre Society on building resilience in vulnerable youth concluded that “[p]ositive relationships provide the most potent protective factors for vulnerable teens” [24]. Research has likewise identified the importance of resiliency in the residential treatment of youth for problematic substance use, such as at the Nimkee NupiGawagan Healing Centre for First Nations youth in Ontario [25].

Positive youth development (PYD) is a multidisciplinary theory that recognizes the strengths of youth and the communities in which they live [26-28]. The goal of PYD is to increase and sustain the positive, healthy development of young people [26], in particular adolescents, and has been applied to programs aimed at preventing substance use [29] (see also Tebes et al., 2007 [30]). The reduction of high-risk behaviour is a core tenant of the PYD framework, recognizing that low risk negative behaviours are a part of healthy youth development [26,31,32]. PYD focusses on three key areas: being universal (targets all youth rather than only 'at-risk’ youth, with the understanding that all youth can benefit from strengthened environments; [26]), systems focussed (youth development is embedded within many integrated, interactive contexts, including family, school, community, society, culture and history [26,32]), and strength-based (views youth as resources to be nurtured, encouraging the growth of inherent capacities for positive development and competence) [27,29,31].

A fourth element of PYD is the promotion of developmental assets, which synthesizes the theory’s systems focussed and strength-based areas into a structured and measurable evidence-base. Exemplified in the Search Institute’s 40 Developmental Assets, the assets are aimed at increasing youth well-being and are often thought of as the 'building blocks’ for healthy child and adolescent development [33,34]. This developmental asset approach is informed by the concepts of resiliency and protective factors, and is based on research pinpointing 20 external and 20 internal assets (see Table 1). These assets have been determined to: 1) prevent risky behaviours, including substance abuse and violence, 2) augment positive outcomes such as school success, and 3) support resiliency in youth [35]. Researchers have found that the more developmental assets a young person has the more positive their development will be, or the less likely they will be to engage in risky behaviours [26,34,36]. For example, in a study assessing the utility of an instrument designed to measure substance use relative to developmental assets (i.e., the Search Institute Profiles of Student Life: Attitudes and Behaviors), it was found that for tobacco, alcohol and illicit drug use, on average 47% of youth in grades 6 – 12 with 0–10 developmental assets either used alcohol three or more times in the past month or got drunk once or more in the past 2 weeks, smoked one or more cigarettes every day or used chewing tobacco frequently, or used illicit drugs three or more times in the past year, whereas on average 2% of youth with 31–40 developmental assets engaged in the same behaviours [35].

**Table 1:** **40 Developmental assets for adolescents (12–18 years of age)**[33]

**External assets**		**Internal assets**	
**Support boundaries and expectations**	**Empowerment constructive use of time**	**Commitment to learning positive identity**	**Positive values**
**Family support**: Family life provides high levels of love and support	**Service to others:** Young person serves in the community one hour or more a week.	**Equality and social justice:** Young person places high value on promoting equality and reducing hunger and poverty.	**Positive view of personal future:** Young person is optimistic about her personal future.
**Positive family communication:** Young person and her parent(s) communicate positively, and young person is willing to seek advice and counsel from parents.	**Other adult relationships:** Young person receives support from three or more nonparent adults	**Integrity:** Young person acts on convictions and stands up for her or beliefs.	**Restraint:** Young person believes it is important not to be sexually active or to use alcohol or other drugs.
**School boundaries:** School provides clear rules and consequences.	**Safety:** Young person feels safe at home, school and in the neighbourhood.	**Homework:** Young person reports doing at least one hour of homework every school day.	**Achievement motivation:** Young person is motivated to do well in school.
**Parent involvement in schooling:** Parent(s) are actively involved in helping the child succeed in school.	**Adult role models:** Parent(s) and other adults model positive, responsible behaviour.	**School engagement:** Young person is actively engaged in learning.	**Planning and decision making:** Young person knows how to plan ahead and make choices.
**Creative activities:** Young person spends three or more hours per week in lessons or practice in music, theatre, or other arts.	**Religious community:** Young person spends one hour or more per week in activities in a religious institution.	**Bonding to school:** Young person cares about her school.	**Reading for pleasure:** Young person reads for pleasure three or more hours per week.
**High expectations:** Both parent(s) and teachers encourage the young person to do well.	**Positive peer influence:** Young person’s best friends model responsible behaviour.	**Caring:** Young person places high value on helping others.	**Honesty:** Young person “tells the truth even when it is not easy”.
**Youth as resources:** Young people are given useful roles in the community.	**Youth programs:** Young person spends three or more hours per week in sports, clubs, or organizations at school and/or in the community	**Interpersonal competence:** Young person has empathy, sensitivity and friendship skills.	**Cultural competence:** Young person has knowledge of and comfort with people of different cultural/racial /ethnic backgrounds.
**Family boundaries:** Family has clear rules and consequences and monitors the young person’s whereabouts.	**Neighbourhood boundaries:** Neighbours take responsibility for monitoring young person’s behaviour.	**Personal power:** Young person feels she had control over “things that happen to me”.	**Sense of purpose:** Young person reports that “my life has a purpose”.
**Caring school climate:** School provides a caring, encouraging environment.	**Community values youth:** Young person perceives that adults in the community value youth.	**Responsibility:** Young person accepts and takes personal responsibility.	**Peaceful conflict resolution:** Young person seeks to resolve conflict nonviolently.
**Caring neighbourhood:** Young person experiences caring neighbourhoods.	**Time at home:** Young person is out with friends “with nothing special to do” two or fewer nights per week.	**Self-esteem:** Young person reports having high self-esteem.	**Resistance skills:** Young person can resist negative peer pressure and dangerous situations.

Positive youth development theory and the 40 developmental assets approach both recognize that specific youth populations may derive unique benefits from protective factors. It is well-established in the literature, for example, that violence and trauma, sexual and physical abuse, stigma and discrimination, and social attitudes and pressures differentially impact females and males [10,37]. The results of two meta-analyses relay that girls may benefit more than male participants from resistance skills training [38,39], programs that reduce social influences to use drugs [39], and altering perceived social norms [39]. There is also research to suggest that females benefit more from competency enhancement or life skills training than do males, however, both derive some benefits [38]. Individual factors have also been shown to be specifically related to drug use and abuse for females in rural communities. For example, one study found that rural female youth who showed aggressive characteristics, who lacked parental support, and who were isolated from peer attachments should be the primary target of substance use prevention programs [40]. Research has also relayed that healthy identity and cultural connections are protective factors for Aboriginal youth and endemic to their well-being [41,42]. Although the 40 developmental assets have not been applied extensively to at-risk Aboriginal youth, some work in Canada has highlighted their applicability [43].

In this paper, we share the 'preptory’ outcomes of our team’s program evaluation of the Prince Albert Parkland Health Region Mental Health and Addiction Services’ Outreach Worker Service (OWS) in eight rural, community schools three years following its implementation. In the province of Saskatchewan, the Ministry of Health supports the application of empirically-based approaches to youth drug prevention services to achieve its goal of “targeting the effective use of child and youth mental health and addictions resources toward improved mental health and well-being functioning for children, youth, and their families” (Saskatchewan Ministry of Health, Community Care Branch [44]). Despite this, and for a myriad of reasons (e.g., funding time constraints), it sometimes happens that provincially-funded programs are delivered prior to the establishment of evidence-informed goals and objectives. This is the case with the OWS.

The paper begins with a brief overview of the OWS program. Next, we detail the establishment of the OWS’s overarching program goals and objectives by our program evaluation alongside the OWS. We did this through in-depth focus groups with the OWS staff, OWS and school administrator interviews, and less intensive focus groups with school staff. With this understanding, we then detail how and why our evaluation team and the OWS staff aligned the program with the Positive Youth Development theoretical evidence-base, emphasizing the program’s universality, systems focus, strength base, and promotion of assets. This included gaining increased clarity about the OWS’s definition of and engagement with its 'at-risk’ student population. The paper concludes with a discussion of how, if time does not permit for the establishment of evidence-informed goals and objectives at the start-up of a program, obtaining insight and expertise from program personnel and school staff and administrators can bring the program to a point where this can be achieved and theoretical linkages made after a program has been implemented. This is a necessary foundation for measuring an intervention’s success.

### Outreach Worker Service

The OWS originated in 1996 in urban high schools in the Prince Albert Parkland Health Region (PAPHR) and was expanded in 2005 into one rural school. PAPHR is located in north central Saskatchewan, and is the third largest of Saskatchewan’s health regions with a population of approximately 80,000, with diversity most prominent by Aboriginal student representation. Motivated by a provincial funding opportunity in 2007, Health Region administrators further expanded the OWS to six rural schools in two PAPHR school divisions in northeastern Saskatchewan that were informally identified as having 'at-risk’ 12–17 year old youth populations. 'At-risk’ was understood as meaning that youth were 'at risk’ both for substance abuse problems and potential co-occurring mental health concerns.

The environment of the communities in which the eight participating rural schools are situated have characteristics that could give rise to high levels of drug use. First, many of the communities experience increased levels of poverty. Many of the parents in these communities work outside of the local community, which limits their parental and school involvement. Excessive alcohol use is highly normalized amongst the adult community populations. And there is a lack of extracurricular activities available to youth, coupled with limited accessibility of the activities that exist due to transportation barriers.

The initial expectations of the OWS were to screen, assess, and work with students on substance abuse and less severe mental health related issues (e.g., mild depression), act as a triage and referral unit for more severe mental health related issues (e.g., autism), and provide standard skills building programming (e.g., group counseling support). According to senior PAPHR Administrators with clinical experience in rural schools, this set up two general expectations for the outreach worker positions: (1) they would be the sole resource for substance abuse/addiction related issues at a given school, and (2) they should attempt to build trusting relationships with all members of the school community for meaningful youth engagement.

Although minimizing risk was the initial focus of the OWS, the role of protective factors was also acknowledged. This coincides with the Saskatchewan Ministry of Education designation of a community school, of which six of the PAPHR schools are officially designated. “Community Schools are [to be] responsive, inclusive, culturally affirming and academically challenging. The learning program and environment effectively build on strengths to address the needs of the communities they serve” (Government of Saskatchewan, Community Schools website). The Ministry promotes the community school philosophy across the province based on its universal value, while recognizing that it is especially meaningful for 'at-risk’ youth.

The eight participating PAPHR schools varied in their access to OWS program staff based on perceived need, with three schools accessing a full time employee, one school accessing one worker four days a week, and four schools accessing one worker approximately one day a week. All of the OWS staff held social work-related skills and competencies to undertake the position. Each school worked in collaboration with the Health Region to set-up its OWS. On the ground, the outreach workers designed their programs based on their school demographics, a strength-based philosophy (e.g., 40 developmental assets), and their individualized professional skill sets. All of the initial expectations of the OWS were addressed (i.e., screen, assess, work with students, referral, standards skills building), and added to during the three years of program implementation: specialty skills-building programming (e.g., wilderness camping), (5) educational services (e.g., classroom education), (6) policy changes and development (e.g., disciplinary role), and (7) community development (e.g., inclusion of family in school activities). Limited attention was allotted throughout to the evidence-based theoretical literature.

## Methods

Data collection and analysis for the preptory stage of our team’s program evaluation focused on: (1) establishing the overarching OWS program goals and objectives across the eight schools, (2) defining the targeted youth student population and OWS’s engagement with them, (3) and identifying and then aligning the OWS with the empirically-informed Positive Youth Development (PYD) theoretical framework. Data collection for all stages drew upon multiple sources, utilizing a mixed-methods approach. Others have found mixed methods to be ideally suited for all stages of program evaluation and specifically to evaluate drug abuse programming [45]. Ethics approval for this study was attained from the two participating PAPHR school districts.

Data were collected in focus group and interview formats, including four in-depth focus groups with the OWS staff (averaged four 180 minutes each), three OWS interviews (two managers and a clinical supervisor) (average 90 minutes each) and nine school administrator interviews (principals, vice-principals and superintendents) (average 60 minutes each), and eight less intensive focus groups with informed school staff (average 90 minutes each; 8 principals, 2 superintendents, and 23 school staff, mainly teachers). Semi-structured interview and focus group guides were developed specific to each of the populations. The guides were structured to facilitate the collection of background information on the start-up of the OWS in each school, patterns of youth substance use, and the scope and nature of the drug prevention service offerings and needs. Data collected through a questionnaire with OWS staff and analysis of existing OWS program data are not presented in this paper because of their septe focus.

All in-depth focus groups and interviews were conducted by the project leads (CAD and CRD) and the less intensive focus groups with the school staff by the research assistants (MB, MS, HT). Notes from the interviews and focus groups were recorded electronically by a note taker, as close to verbatim as possible. The data were analyzed by the project leads using a manual, general inductive approach. The coding process involved recognizing important points/statements, from which categories were developed, and the findings subsequently interpreted. Our choice of an inductive approach allowed the findings to emerge from the objectives of the preptory stage of our program evaluation. We undertook coding consistency checks through independent pllel coding measures and applied stakeholder checks to our interpretations and findings with the OWS staff and administration. A general inductive approach was chosen because it “…provides an easily used and systematic set of procedures for analyzing qualitative data that can produce reliable and valid findings” [46] (237). We did not choose a more complex qualitative analytic approach (e.g., grounded theory, phenomenology, discourse analysis) because of the specific evaluation aim of the study [47-49].

## Results

### Outreach Worker Service goals and objectives

Applying our choice of methods, the overarching goals (see Outreach Worker Service goals) and objectives (see Outreach Worker Service objectives) of the OWS were established across the eight schools. Although at first it appeared that each school may have been unique in the service it offered because of varying programming activities, the four in-depth focus groups held with the OWS staff, totaling more than 12 hours, relayed that this was not the case. Using a workbook designed to assist organizations with no or little program evaluation experience as a guide (*A Community-Based Workbook for Evaluating Substance Abuse & Mental Health Programs in Saskatchewan*[50]), two well-defined goals and four corresponding objectives were developed for the OWS. They support the original and expanded expectations of the OWS during its three years of operation, and were verified in interviews with OWS and school administrators and focus groups with the school staff.

### Outreach Worker Service goals

The goals of the OWS are:

1. To improve the wellbeing of youth, schools and communities by increasing youths’ 40 developmental assets to prevent/minimize the problematic use of alcohol and other drugs and substances and related mental health concerns with four groups of youth through building therapeutic relationships, asset development and application, education, and community development.

2. To be part of the culture of the school by connecting with youth, families, the school and broader community. This includes community development, recognizing that communities within the Outreach Worker Service are at different stages of engagement.

### Outreach Worker Service objectives

The objectives of the OWS are to:

1. Build therapeutic relationships by

i) engaging at risk students through an orientation led by the outreach worker

ii) supporting the counseling of at-risk students by engaging caregivers when relevant

iii) applying the appropriate therapeutic tools to increase student wellbeing

iv) recognizing client needs and determining appropriate referral services as necessary

v) collaborating with school staff on a student’s therapeutic process as appropriate

vi) providing support services to community members as requested (and as time permits)

2. Develop and apply students’ assets by

i) developing and delivering life skills programming (i.e., 40 developmental assets)

ii) providing opportunities to apply/practice life skills (experiential learning)

3. Offer education by

Youth

i) educating student clients (which includes their families) in one-on-one mental health and substance abuse related issues, as identified through an individual assessment session

ii) educating students in one-on-one and classroom group sessions

iii) acting as an informational and educational resource to students and families

Staff

i) serving as a resource to staff to meet their health curriculum needs

ii) serving as a resource to staff to meet student needs as identified by staff and/or outreach workers

Community

i) educating the community on promoting student health

4. Develop community (it takes a whole village to raise a child philosophy) by

i) Supporting community opportunities for access to and growth of services by promoting connectedness and well-being.

### 'At-risk’ youth

Intrinsic to discussions during the in-depth OWS staff focus groups was a recurring and complex dialogue regarding the program’s target population, originally defined as youth between 12–17 years of age and 'at-risk’ of substance use and dependence. There was variability amongst the OWS staff in defining 'at-risk’ youth, which mirrors the discrepancy found in the research literature [51-53]. There was underlying agreement, however, about the circumstances that place some youth 'at greater risk’ for problematic substance use, primarily individual/personal, interpersonal (family, peers, school), and social and cultural/community factors.

Three additional themes emerged from the OWS staff focus groups regarding the definition of youth as 'at-risk’. First, it was felt that there was divergence between the strength-based focus of the OWS and the deficit based focus of defining an 'at-risk’ youth population. Second, the OWS staff were unwilling to place students in dichotomous categories of either 'at-risk’ or 'not at-risk’, and hence their preference for a tiered approach including 'low risk’, 'moderate risk’ and 'high risk’ to use and abuse substances [51]. However, despite their desire to not use a dichotomous categorization, program staff indicated that such a classification was necessary for service delivery. For example, identifying students as 'high-risk’ in an assessment helps to ensure that their services are prioritized. Moreover, mental health problems and issues concerning general well-being (e.g., youth having trouble at home, youth coping with parental break-up, peer issues like bullying, problems with self-image, among others) were identified as important markers for being 'at-risk’ of substance use and dependence. Finally, the impacts of the social determinants of health (e.g., education, social support, gender, social economic status) were also recognized in the focus groups.

It is important to note as well that the factors placing youth 'at-risk’ and mentioned most prominently in the OWS staff focus groups were similarly identified in the semi-structured interviews with OWS and school administrators and the focus groups with school staff. They highlighted their rural specificity, including social economic status (foremost poverty but also high economic status associated with rural economies due to farming income and access to funds to purchase substances), familial issues, such as experiences of violence and abuse and single parenting (including disruption with typically a father away at work for extended periods of time), younger kids associating with older kids (rural schools are K-12), and event-specific opportunities, such as rural bush parties. One principal commented: “Certain events and time of the year have heavier use, especially of alcohol (e.g., sporting tournaments, graduation)… [There is] [s]ome torrid history of this within the health region. Parental supervision may be involved in approving alcohol use at an event” (School Principal, Interview #6).

Based on this understanding, four 'at-risk’ student groups were identified by the OWS workers and verified in the follow-up school administration interviews and school staff focus groups (see Outreach Worker Service 'At-risk’ youth). The categories are hierarchically ranked, indicating the OWS staff concentrated their work based on identified need with the 'high-risk’ students, followed by mental health, general prevention and then crisis situations. A school staff member succinctly shared their general insight and support for such a comprehensive definition of 'at-risk’ youth: “The four categories are specific but also broad, and they would include everyone, and no one would fall through the cracks” (School Staff, Interview #21).

### Outreach Worker Service 'At-risk’ youth

1. High risk youth who are not using substances (high risk meaning low on developmental assets).

High risk youth using substances

High risk youth abusing substances (e.g., binging on the weekend)

2. Mental health issue related youth not using substances (e.g., depressed youth and used cannabis once, as an example; this category includes self-harm potential)

Mental health issue related youth using substances

Mental health issue related youth abusing substances

Mental health issue related youth who specifically have FASD (may or may not be using/abusing substances)

3. Low risk at present youth (prevention focus with these youth)

This category may also include pre-mental health issue related youth (for example, have low social/life skills)

Male and female students were identified by the OWS and school staff and administration as being at equal risk, with acknowledgement that they may face different risk factors. For example, patriarchal gender roles are well established in rural communities and can have a detrimental influence on girls’ lives. Teen pregnancy was identified as an associated concern. Little to no attention was paid to other diversity factors, such as gender identity and sexual orientation. Some attention was paid to cultural diversity, and specifically to Aboriginal youth. Aboriginal youth were identified as a specific 'at-risk’ population, with schools having a higher Aboriginal student population identifying a higher incidence of economic disadvantage, foster care involvement, issues with school attendance and transiency. It was also relayed that there is a general lack of integration between First Nation reserve and non-reserve students. All schools indicated an effort (although to varying degrees) to include Aboriginal worldviews and cultural practices in their curriculum, with those schools receiving funding as a community-designated school being required to provide Aboriginal contextualized curriculum and to be culturally affirming.

### Positive youth development

Drawing on expert knowledge is important for the development of youth drug prevention programming, but attention must also be paid to aligning and coordinating professional health care services with a theoretically informed evidence-base for evaluation purposes. It is important for mental health and addictions programming to link with theoretical constructs as they are considered essential for measuring intervention success and conducting program evaluation studies [54]. Grounded in a strength based approach, the PYD framework was identified from the theoretical literature as a potential evidence-base for the OWS. A systematic comparison of the OWS with PYD revealed an amicable fit, noting their near identical recognition of universality, a systems focus, a strength base, and the promotion of assets.

The OWS supports the **universal** element of PYD theory; that is, targeting all youth rather than only 'at-risk’ youth, with the understanding that all youth can benefit from strengthened environments. The OWS targets all youth in the student population, in contrast to only a group of 'at-risk’ or 'at high risk’ youth. As relayed, the OWS identifies all youth on a continuum of risk for substance use and related mental health concerns. For example, youth identified as being at 'low-risk’ are targeted with early prevention efforts. This includes classroom education sessions, life skills programming to develop and strengthen assets, and having access to the OWS worker as an informational and educational resource. As a further example, all students (high and low risk) potentially benefit from the strengthened context of the community and resources available within the school because of the OWS’s presence. Nevertheless, it should be noted that some high-risk classified youth do necessarily receive more specialized services (i.e., family counselling, crisis intervention, etc.). Also, diversity is acknowledged within the universal framework of PYD and the OWS, for example, it is recognized that some student populations, such as male and female students as well as Aboriginal students, are affected by unique risk factors and therefore these must be addressed in response.

A **systems** view is explicitly supported within the OWS through its focus on the environment and supports surrounding youth, specifically the school and immediate community. PYD proposes that youth development is embedded within many integrated, interactive contexts, including family, school, community, society, culture and history. The OWS is designed on a philosophy of increasing student health while simultaneously increasing school and community well-being, as outlined in the OWS goals and objectives and promotion of the adage that “it takes a village to raise a child” [55]. Family is also clearly identified as a part of the system by the OWS, evident in the OWS’s inclusion of family members in counselling sessions and staff efforts to build relationships with school, family and community members. Finally, the OWS recognizes that for the youth population it is serving, the systems-related determinants of health impact many students’ well-being and 'at-risk’ status, and therefore must also be recognized.

PYD supports a **strength-based framework** that views youth as resources to be nurtured, encouraging the growth of inherent capacities for positive development and competence. Similarly espoused by OWS is the view that youths’ inherent capacities for healthy well-being need to be fostered. A strength-based framework is explicit in the goals of the OWS to increase youth, school, and community well-being, in light of the fact that it is a prevention and intervention program for substance use and mental health concerns. In other words, the goal to prevent and/or minimize substance abuse is intertwined with expected improved general well-being. Thus, the OWS focuses on increasing and strengthening positive attributes (developmental assets, resiliency), rather than eliminating or preventing negative attributes (substance use). This fits with the PYD framework’s recommendation that negative outcomes can be reduced by strengthening positive assets.

With the strength-based focus of the OWS, and staff awareness of the 40 developmental assets, this was the natural overlapping point between the OWS and PYD’s promotion of **assets**. The merits of the 40 developmental assets were identified foremost for their strength-based foundation and ability to concurrently address students (personal issues), families (family dynamics) and the community (community engagement and linking to services and supports). The OWS placed specific emphasis on three assets: self-esteem, developing a sense of purpose/future, and role modeling. Although not consistently referred to as the 40 developmental assets by the OWS, they are a foundation to the program’s development.

## Discussion

Adding a 'preptory’ stage to our team’s program evaluation of the OWS enabled us to identify the goals and objectives of the OWS and define its targeted 'at-risk’ student youth population three years after the program’s initial implementation. This is in line with how programming is established in the community at times, including Saskatchewan, often with insufficient time and resources to identify measurable program goals and objectives at the outset [56,57]. For example, a study by Carman (2007) shared that few organizations that deliver programming have either the time or funds required to carry out an evaluation study, no matter how important they believe it to be (e.g., to relay to funders their success to maintain programming funding) [58]. Our team’s approach also enabled us to identify a theoretically informed evidence-base for the OWS. This, alongside clearly defined goals, objectives and a target audience, is essential for the eventual undertaking of a complete program evaluation process. Work in this area, as illustrated in a case study by Larsen, Tax and Butock (2009), supports the important role of evaluation in moving organizations forward, including unifying their service delivery [57].

The OWS’s definition of its **goals and objectives** is supported in the literature on effective substance use prevention in youth and schools. The goals and objectives generally coincide with the empirical literature indicating the effects of protective factors in reducing the impact of risk factors for substance use. The overarching OWS’s goals and objectives reflect the protective factors of building community assets, strengthening individual resilience [12,16,25], forming connections to school [59] and forming positive relationships with others, including caring adults [24].

More specifically, the OWS’s emphasis on improving well-being (goal 1) reflects the strength-based approach supported in the literature [60]. Including a focus on at-risk students is likewise supported in the literature, with specific positive outcomes identified for targeted interventions with at-risk students [61].

Offering life skills programming to students is another key feature of the OWS (goal 1 & objective 2) that researchers have shown to improve outcomes [62]. The most effective services are those that use cognitive behavioral programming, such as life skills development [61-63]. In addition, the use of experiential activities for students to apply and practice life skills in an interactive delivery method has been shown to reduce substance use [61] and has been identified as a best practice at-risk approach [62].

Inherent in the OWS’s goals and objectives is a focus not only on students, but also families, schools and communities, such as increasing well-being (goal 1), connecting with additional groups (goal 2), as well as offering education (objective 3) and investing in community (objective 4). In the literature, such a holistic approach that includes multiple factors and emphasizes building collaborative relationships is supported as a best standard/practice for substance use prevention in youth [60] and for school-based programs [29].

The expanded **definition of 'at-risk’ youth** by the OWS is likewise supported in the current literature, where definitions of youth 'at-risk’ for substance use vary. Indeed, some researchers apply the term 'at-risk’ to include students who are at “low risk”, “moderate risk” and “high risk” to abuse substances [64], although the emerging trend is to recognize student diversity and not categorize all students as one and the same [5,29,53,65].

The risk factors identified by the OWS correspond in large part to the common overriding risk factors discussed by previous researchers (e.g., negative risk taking tendencies [2], poor psychological health [2], limited school and community bonding [15,16]). It is also important to note that the OWS’s recognition of the social determinants of health in combination with risk factors for youth substance abuse helps to more precisely define, understand and respond to who 'at-risk’ youth are.

The loose theoretical linkage in developing the OWS to the 40 developmental assets framework amongst the majority of the school sites is a key strength in its delivery of effective programming. Generally, when a program is based on an explicit theory and it is understood and used to design the activities of the program, it results in a more positive reduction of substance use or abuse [61]. This became even more explicit with alignment of the OWS to the **Positive Youth Development** theoretical evidence-base, emphasizing the program’s universality, systems focus, strength base, and asset promotion.

According to the Health Canada (2001) compendium of best practice guidelines for preventing substance use problems among young people, there are numerous characteristics of effective programming and they consistently emphasize a strength-based approach [60]. These include promoting youths’ strength of resiliency, using a holistic approach (systems approach, including individual, school, family, and community factors), incorporating skill development as a main component, and intensifying the program as the risk of the youth increases. Health Canada’s best practice guide suggests that as youth experience more risk factors, they are more likely to use substances, as well as engage in other problematic behaviours. The strengths of the youth are in a constant interaction with their risk factors. It follows that if protective factors can be strengthened, engagement with risky behaviours may decrease [60].

The focus on protective factors and resiliency alongside risk factors (a strength-based approach) is identified as a best strategy for positive behavioural and attitudinal outcomes [29,60]. Focusing on youths’ capabilities and strengths rather than their limitations or deficits, influences youth to respond to programs in a more positive way. With this approach youth are active agents in their development, which helps to further foster protective factors. Some of the key protective factors that the 2010 Canadian School Standards identify are competence, self-confidence, connectedness, character, caring, compassion, and safe and welcoming environments [29]. This strength-based approach, alongside the 2001 Health Canada best practice guide, coincide with the OWS’s application of the 40 developmental assets and more specifically its adherence with the Positive Youth Development framework; both focus on positive youth development as opposed to being problem or deficit-based.

Developmental psychology researchers offer evidence for the effects of assets and PYD components in promoting healthy adolescent adjustment. It has been demonstrated that the presence of a high number of developmental assets is related to low levels of substance use [35]. An example of a PYD program designed to prevent problematic substance use is the Positive Youth Development Collaborative, which teaches youth prevention skills and health education through culturally relevant activities. The main component of this prevention program focuses on effective decision making, coping with stress, information about substances, and applying decision making to one’s own life. Results of an evaluation with mainly African American and Hispanic participants in the United States indicated that students who received the intervention were more than six times as less likely to use alcohol than the control students, and they were also less likely to use marijuana as compared to the control students. The students in the intervention were also less likely one year after the intervention to use alcohol, marijuana, or other drugs than the control students. Although there was still an increase in substance use as the intervention participants aged, it was significantly less than the increase with the control students [30].

Upon reflection, the OWS staff and administrations, with the guidance of our independent evaluation team, identified three key insights through their participation in the 'preptory’ stage of a program evaluation. First, they came to a clear understanding that it was necessary to undergo this preptory stage to eventually conduct a program evaluation of the OWS, to which they are committed. They shared how, in future program development, they will also be able to transfer this knowledge and its importance. Second, the staff recognized how similar they are in the OWS programming they offer, even though they are at different school locations and with somewhat varying populations. Identifying the overarching program goals and objectives made this clear. Similarly, developing a definition of the 'at-risk’ student population relayed how similar they are implementing the OWS program. And third, the OWS staff were able to clearly identify how they expanded the OWS program expectations since its start-up, as originally identified by the OWS administration. In fact, the absence of overarching goals and objectives may have allowed the program greater flexibility in its continued development and response to student-identified needs. It was important to document this for future evaluation purposes, as well as a reminder to the OWS staff to better information share amongst themselves and with their administration.

### Limitations to the current study

There are two key limitations to the current study. The first is that a 'comprehensive’ program evaluation could not be completed because of the necessary upfront work that needed to be undertaken, including identifying the OWS goals and objectives, defining 'at-risk’ youth, and linking the OWS to an empirically-informed theoretical framework, which in this case was PYD. The next step for the OWS will be to undertake a process evaluation with our evaluation team, commonly defined as “[t]he systematic collection of information on a program’s inputs, activities, and outputs, as well as the program’s context and other key characteristics” [66]. The evaluation work completed to date will be beneficial to addressing the overall goal of a process evaluation, which is to document what is happening in a program and recognizing, as noted in a tobacco control process evaluation, that “the type of information gathered, and its frequency, will depend on the kinds of questions that you seek to answer” [66]. The outcome of the process evaluation in our work is an important step forward in establishing the groundwork to link progress and activities to outcomes in a future outcome evaluation.

Second, the data collected in this study is not generalizable beyond the PAPHR in Saskatchewan, Canada. However, the documentation of the research process and content is a significant contribution to the literature and to others undertaking program evaluations; it is necessary to document the difficulties and solutions to doing research in the real-world [56]. Similarly, Dykeman et al. (2003) wrote an academic article on the development of a program logic model to measure the processes and outcomes of a program “because we found limited descriptions of the process in the literature” (197) [67].

## Conclusions

This paper shared the 'preptory’ outcomes of our team’s process evaluation of the Prince Albert Parkland Health Region Mental Health and Addiction Services’ Outreach Worker Service (OWS) in eight rural, community schools three years following its implementation. It was necessary to begin at this preptory stage because as sometimes happens, programming was delivered prior to the establishment of evidence-informed goals and objectives. Our team helped to establish the overarching program goals and objectives, and its targeted definition and engagement with an 'at-risk’ student population. This, in turn, enabled us to align the OWS with the empirically-informed Positive Youth Development (PYD) theoretical framework, acknowledging the overlap between it and the OWS’s attention to developmental assets. Through this process we concluded that undertaking a process evaluation three years after the OWS’s initial implementation was possible. In fact, this time frame may have allowed the program greater flexibility in its continued development and response to student-identified needs. Nonetheless, this is a necessary foundation for measuring an intervention’s success. This is an important contribution to the literature and for others trying to undertake programming research in the 'real world’.

## Abbreviations

OWS: Outreach Worker Service; PAPHR: Prince Albert Parkland Health Region; PYD: Positive Youth Development.

## Competing interests

All authors declare they have no competing interests. CM and BE declare they work for the Prince Albert Parkland Health Region.

## Authors’ contributions

CAD and CRD participated in the design of the study, performed the data analysis, and helped draft the manuscript. CM and BE conceived of the study and participated in the study design. TQ participated in the study coordination and literature review. MB, MS, and HT participated in the data collection and data analysis. AD helped draft the manuscript. All authors read and approved the final manuscript.
